# Clustering of children's activity behaviour: the use of self-report versus direct measures

**DOI:** 10.1186/1479-5868-8-48

**Published:** 2011-05-25

**Authors:** Travis J Saunders, Stephanie A Prince, Mark S Tremblay

**Affiliations:** 1 Healthy Active Living and Obesity Research Group (HALO), Children's Hospital of Eastern Ontario Research Institute, 401 Smyth Road, Ottawa, ON, K1H 8L1 Canada

## Abstract

While we concur with the objectives of the recent *International Journal of Behavioural Nutrition and Physical Activity *paper published by Jago and colleagues titled "Physical activity and sedentary behaviour typologies of 10-11 year olds", we feel that the results as currently presented do not support their conclusions. Though the authors created groups of children with dramatically different patterns of self-reported physical activity and sedentary behaviour, an inspection of the objectively measured accelerometry data shows little difference between the groups. Further, in at least one instance the difference between groups was of the opposite direction when using objective measures, as opposed to the self-report measures used in the published analysis. Thus, we caution the authors from making conclusions based on their self-report data, and propose that they re-analyze their data using their objectively measured data instead.

## 

To the Editor,

We read with great interest your recently published study by Jago and colleagues [1] titled "Physical activity and sedentary behaviour typologies of 10-11 year olds". The authors argue convincingly that interventions which aim to promote increased physical activity and/or reduced sedentary behaviour should focus on the specific needs and characteristics of their target populations. As such, we concur that their objective to identify clusters of children with similar patterns of physical activity and sedentary behaviour would provide key information for the design of targeted interventions. Unfortunately, we believe that the data presented in the paper suggests that the clusters created by the authors do not represent groups of children with distinct activity patterns, and that the conclusions of the paper are therefore unsupported.

In their paper, Jago et al. [1] assessed physical activity and sedentary behaviour using both self-report questionnaires and accelerometry. However, when creating clusters of children with similar behaviour, the authors relied on only the self-reported data. While this resulted in clusters of children with very distinct quantities of self-reported physical activity and sedentariness, the groups appear almost identical when compared using the objectively measured data. For example, according to the self-report data, the "High Activity/Low Sedentary" group performed an average of 3.6 hours more weekday physical activity than children in the "Low Activity/Medium Sedentary" group. However, when the accelerometer-derived values of weekday moderate- to vigorous-intensity activity are compared instead, the difference between the two groups is reduced to roughly two minutes. Thus, in this situation, the difference between the two groups using self-report measures was roughly 100 times greater than the measured difference assessed using accelerometry.

A similar problem is observed when comparing the groups for sedentary time. For example, the self-report data suggests a dramatic difference in screen time (excluding school-work) between the "High Activity/High Sedentary" group which accumulated 13.86 hours per day and the "High Activity/Low Sedentary" group which reported just 5.77 hours per day. In contrast, the objectively measured data suggests that the "High Activity/High Sedentary" group accumulated 4.7 hours of weekday sedentary time outside of class time (roughly 9 hours less than suggested by their self-reported screen-time), and only differed from the "High Activity/Low Sedentary" group by 5 minutes. Similarly, the "High Activity/High Sedentary" group actually accumulated *less *objectively-measured sedentary time than the "Low Activity/Medium Sedentary" group on both weekdays and on weekends. Further, it is questionable whether it would even be possible for children to accumulate the daily volume of screen time (13.86 hours) and physical activity (5.89 hours) reported by children in the "High Activity/High Sedentary" cluster. If true, this would leave the children less than 5 hours per day for both school-work and sleep, suggesting that these values are not just unlikely but impossible.

The large discrepancies between objective and self-report activity patterns observed in the present study have also been reported by others. For example, a recent systematic review by Adamo and colleagues [2] reports that, in comparison to accelerometry, self-report measures overestimate physical activity by an average of 114% in boys and 584% in girls. Recent findings also suggest that self-reported screen time is only weakly correlated with objectively measured sedentary behaviour in adults [3]. It has also been noted that few of the studies which purport to assess sedentary behaviour have actually measured it [4]. Given the discrepancies between self-report and direct measures of activity in the literature, and the availability of directly measured data in the present situation, we caution the authors from making conclusions based on their self-report classifications. Further, we would be interested to know how the behaviour clusters created in the present study might differ if they were based on the accelerometry data, and whether this might also result in more pronounced differences between the clusters in terms of body mass index or the Index of Multiple Deprivation score.

We welcome comments from the authors of the current study in order to provide further clarification of the methods employed and conclusions made.

Respectfully,

Travis J. Saunders, Stephanie A. Prince and Mark S. Tremblay.

## Competing interests

The authors declare that they have no competing interests.

## Authors' contributions

TJS, SAP and MST conceived of the letter, and participated in its design. TJS and SAP participated in the drafting of the manuscript, and MST critically revised it. All authors read and approved the final manuscript.
